# Computational hemodynamic assessment of axillary and femoral artery perfusion for extracorporeal left ventricular assist device

**DOI:** 10.3389/fcvm.2025.1631144

**Published:** 2025-12-02

**Authors:** Shilin Wang, Qianwen Liu, Ping Li, Changdong Zhang, Wenqian Wu, Hao Liu, Peiwen Yang, Lingyun Fang, Wei Su, Jamshid H. Karimov, Nianguo Dong, Shu Chen

**Affiliations:** 1 Department of Cardiovascular Surgery, Union Hospital, Tongji Medical College, Huazhong University of Science and Technology, Wuhan, China; 2 Department of Technology, Boea Wisdom (Hangzhou) Network Technology Co., Ltd., Hangzhou, China; 3 Department of Ultrasound Medicine, Union Hospital, Tongji Medical College, Huazhong University of Science and Technology, Wuhan, China; 4 Hubei Province Key Laboratory of Molecular Imaging, Wuhan, China; 5 Department of Vascular Surgery, Traditional Chinese and Western Medicine Hospital of Wuhan, Tongji Medical College, Huazhong University of Science and Technology, Wuhan, China; 6 Research Collaboration Group, Cardiovascular Innovation and Research, Cleveland, OH, United States

**Keywords:** heart failure, temporary circulatory support, extracorporeal, left ventricular assist device, percutaneous, right axillary artery, computational fluid dynamics, hemodynamics

## Abstract

**Objectives:**

Traditionally, the arterial blood perfusion tube for an extracorporeal left ventricular assist device (LVAD) is placed in the femoral artery, which limits patients' mobility and increases the possibility of lower limb ischemia. We have also developed a new approach for percutaneous placement using the right jugular vein and axillary artery. However, there hasn't been a direct hemodynamic comparison between axillary and femoral perfusion. Therefore, this study estimated the hemodynamic differences between axillary artery and traditional femoral artery perfusion for LVAD.

**Methods:**

Five patients underwent LVAD implantation through the right axillary artery and jugular vein approach at Wuhan Union Hospital. Based on one of their computed tomographic angiography (CTA) data, we established a computational fluid dynamics model. Key hemodynamic parameters, including time-averaged wall shear stress (TAWSS) and oscillatory shear index (OSI), were estimated to be compared.

**Results:**

The axillary perfusion improved perfusion to critical arteries and maintained more stable blood flow distribution, regardless of whether the LVAD supplied 40% or 60% of the total cardiac output. Femoral perfusion caused a substantial reduction in TAWSS and an increase in OSI, suggesting greater blood flow disturbances. In contrast, axillary perfusion maintained TAWSS values closer to normal and exhibited lower OSI, particularly in the thoracic and upper abdominal aorta.

**Conclusions:**

The axillary approach may offer superior hemodynamic performance compared with the conventional femoral approach for percutaneous LVAD, including improved perfusion of abdominal aortic branches and steadier blood flow changes. The jugular-axillary approach could be a promising procedure for future percutaneous LVAD.

## Introduction

Extracorporeal left ventricular assist device (Extra-LVAD) is a standard temporary circulatory support device for patients with severe heart failure, whether transitioning to heart transplantation or transitioning to heart repair surgery (1–3). We have developed a new approach using the right jugular vein and the axillary artery (AA) for percutaneous placement to avoid the disadvantages caused by traditional femoral artery (FA) perfusion, as we previously described in a case report (4). Except for our case, some centers used axillary artery perfusion (5–7). However, a direct hemodynamic comparison of LVAD perfusion between the AA and FA approaches has not previously been reported. In this study, based on the computed-tomographic angiography (CTA) data of a male patient with severe heart failure, we established a computational fluid dynamics (CFD) model. We compared hemodynamics during LVAD perfusion using the AA and FA approaches.

The wall shear stress (WSS), a fluid dynamics concept, describes the frictional force a fluid exerts on a solid wall it contacts. Pathologically, changes in WSS can affect vascular endothelial gene expression or solute transport, such as oxygen and low-density lipoprotein (LDL), near the wall, possibly leading to vascular diseases (8–10). Two parameters, time-averaged WSS (TAWSS) and oscillatory shear index (OSI), help characterize WSS. TAWSS averages WSS over a cardiac cycle. Meanwhile, OSI, a dimensionless parameter ranging from 0 to 0.5, measures the direction variability of WSS and indicates steady to highly fluctuating flow. Our study mainly focused on the characteristics of each aorta part.

## Materials and methods

### Study population

This study was conducted under a protocol approved by the Institutional Review Board of Union Hospital, Affiliated with Tongji Medical College, Huazhong University of Science and Technology, Wuhan, China (number: 2023-0429, approval date: June 6, 2023).

The study protocol complies with the ethical principles of the 1975 Declaration of Helsinki. From May 2022 to January 2023, five patients in our center underwent Extra-LVAD placement via the right jugular vein and AA. We used aorta CTA data from one of these patients.

### 3D Model reconstruction

The contrast-enhanced CTA images were obtained from a heart failure patient with an LVAD. CTA imaging was performed using a dual-source CT scanner (SOMATOM Force, Siemens Medical, Germany, Software Version VA50). Volumetric datasets were retrospectively reconstructed using a multi-segment algorithm, generating thin-section images with a slice thickness of 0.3 mm.

The segmentation region encompassed the aortic lumen and its major branches, extending to the right axillary and femoral arteries as potential LVAD implantation sites. Anatomical model reconstruction was performed through manual segmentation and subsequent smoothing using the open-source software 3D Slicer (Version 5.3.0, National Institutes of Health, USA). The resulting vessel wall model was clipped, and inlet and outlet boundaries were generated to cap the ends, thereby defining a continuous fluid domain for computational simulation.

### Computational method

Blood was modeled as an incompressible Newtonian fluid with a density of 1,050 kg/m^3^ and a dynamic viscosity of 0.0035 Pa·s. The Reynolds-averaged Navier-Stokes (RANS) equations were solved using the Shear-Stress Transport (SST) k-*ω* turbulence model. This hybrid model was selected for its demonstrated efficacy in predicting flow separation under adverse pressure gradients (11), which is particularly relevant for capturing the jet flow dynamics induced by LVAD insertion, and its ability to accurately resolve near-wall flow, which is critical for reliable Wall Shear Stress (WSS) calculations (12). The model integrates the robustness of the k-*ε* model in the far-field with the precision of the k-*ω* model in near-wall regions.

The governing continuity (1) and momentum (2) equations are:

$$\begin{array}{c} \nabla \cdot u\;=0 \end{array}$$ (1)

$$\begin{array}{c} \frac{\partial u}{\partial t}+(u\cdot \nabla )u=-\frac{1}{\rho }\nabla p+\nu \nabla^{2}u \end{array}$$ (2)

Where *ρ* is the fluid density, u is the velocity vector field, p is the pressure, ν is the kinematic viscosity.

The spatial discretization employed a second-order upwind scheme, and the pressure-velocity coupling was handled using the Semi-Implicit Method for Pressure-Linked Equations (SIMPLE) algorithm. Solutions were considered converged at each time step when the maximum normalized pressure residual fell below 10^−4^. The fluid domain was discretized using a hybrid mesh, comprising tetrahedral elements in the core and three layers of prismatic elements adjacent to the wall to capture the boundary layer (Supplementary Figure S1) accurately.

A grid independence study was conducted to ensure robust results (Supplementary Figure S2). Simulations were performed on coarse (∼1 million cells), medium (∼2.2 million cells), and fine (∼5 million cells) meshes for the baseline model (without LVAD). The difference in computed WSS between the medium and fine meshes was less than 1%. Consequently, the medium-resolution mesh was adopted for all subsequent simulations (approximately 2.2 million cells for the baseline, and 2.4 million for LVAD-supported models). The near-wall resolution was controlled to achieve a y^+^ value of approximately 1, consistent with the SST k-*ω* model's requirements.

The time step was fixed at 1 ms to ensure temporal accuracy. Simulations were run for three cardiac cycles to achieve a periodic solution, with data from the final cycle used for analysis. The aortic wall was modeled as rigid with a no-slip boundary condition. The simulations were performed using OpenFOAM (version 11).

**Figure 1:**
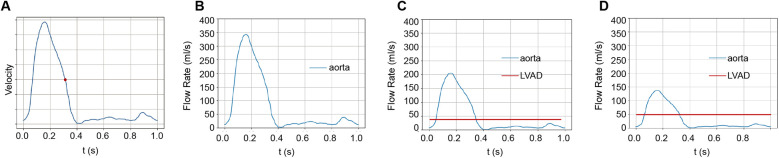
The origins of blood flow during a cardiac cycle. **(A)** We studied blood flow velocities at the end-systolic phase, marked by the red dot during the cardiac cycle. **(B)** In the standard group, the heart propels all blood. In this study, we assumed a cardiac output of 5 LPM. **(C)** With LVAD supplying 40% of total flow, 2 LPM of blood is perfused from the LVAD and 3 LPM from the heart. **(D)** With LVAD supplying 60% of total flow, 3 LPM perfused from LVAD and 2 LPM from the heart.

**Figure 2:**
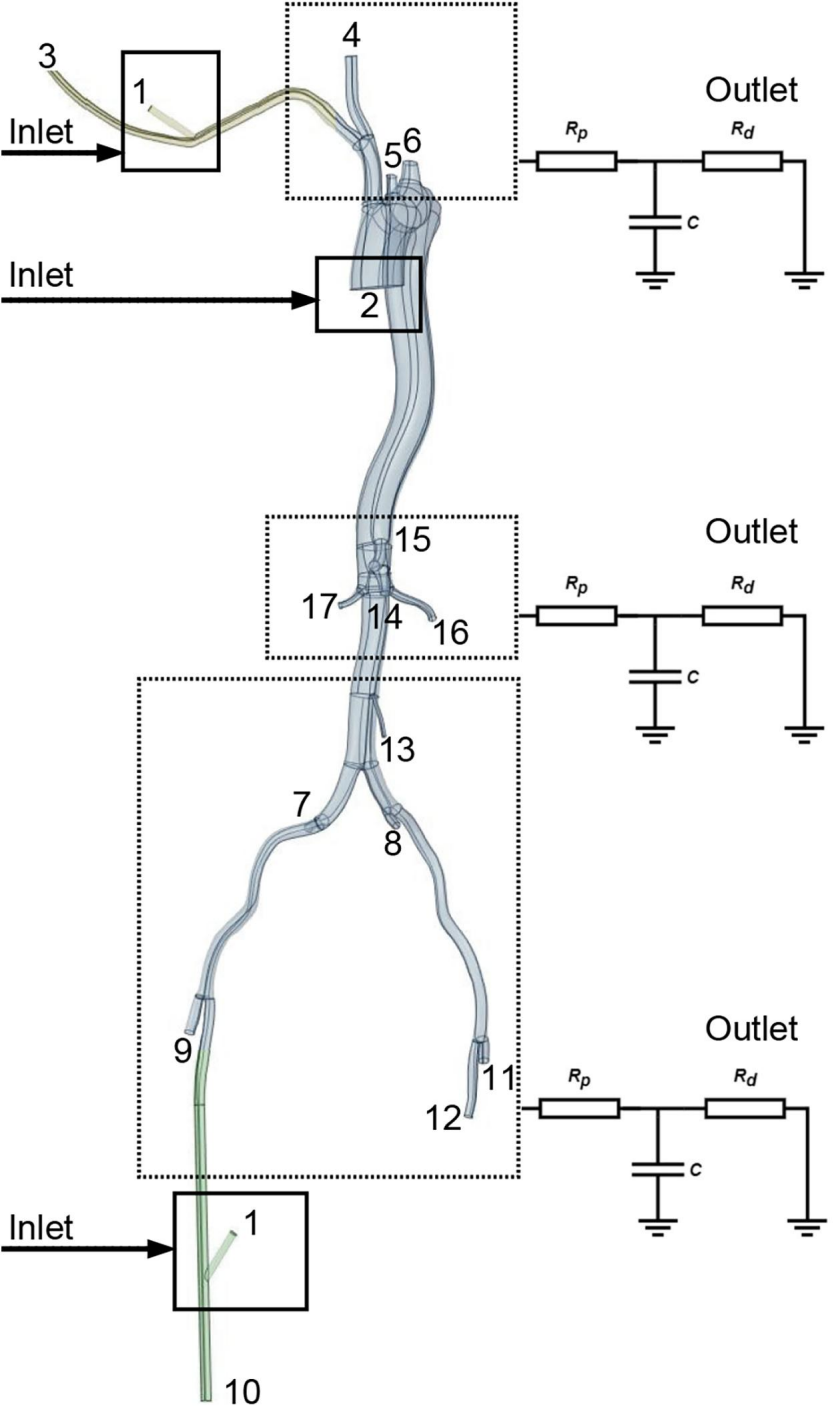
Identification of specific aortic branch locations referenced in Tables 1, 2. Points 1 and 2 signify the LVAD and left ventricle inflow locations. The square denotes the location of the graft for perfusion. Points 3-12 indicate the perfusion outlet sites for each aortic branch, calculated using Windkessel models.

A physiologically representative flow waveform for the ascending aorta, corresponding to a cardiac output (CO) of 5 L/min for a healthy individual (13), was prescribed at the inlet (Figure 1A). To model the impedance of the peripheral vasculature, a tightly coupled 0D-3D scheme was implemented within OpenFOAM. This scheme solves the three-element Windkessel (0D) model equations in a tightly coupled manner with the 3D Navier-Stokes equations at each time step. A three-element Windkessel (0D) model, characterized by proximal resistance (R_p_), compliance (C), and distal resistance (R_d_) (14), was applied at each outlet (Figure 2, Supplementary Figure S3). The Windkessel parameters were calibrated to match reference systolic/diastolic pressures and presumed flow splits under normal conditions (without LVAD support).

**Table 1:** Flow divisions in aorta branches with and without LVAD, considering femoral and axillary perfusion at 40% of the total flow.

Branch of aorta^a^	Without LVAD	LVAD via femoral artery perfusion		LVAD via axillary artery perfusion	
	Flow division (%)	Flow division (%)	Variation (%)	Flow division (%)	Variation (%)
Input					
LVAD inflow (1)	0%	24.7%	-	24.7%	-
CO (2)	100.0%	75.3%	-	75.3%	-
Output					
Upper extremity arteries and carotid arteries					
Right subclavian artery (3)	3.0%	2.8%	−0.2%	3.2%	0.2%
Right common carotid artery (4)	7.3%	7.0%	−0.3%	7.2%	−0.1%
Left common carotid artery (5)	4.3%	4.4%	0.1%	4.6%	0.3%
Left subclavian artery (6)	9.2%	9.3%	0.1%	10.1%	0.9%
Lower extremity arteries					
Right internal iliac artery (7)	3.9%	3.6%	−0.3%	3.8%	−0.1%
Left internal iliac artery (8)	3.9%	3.6%	−0.3%	3.8%	−0.1%
Right profunda femoris artery (9)	5.3%	4.9%	−0.4%	4.9%	−0.4%
Right femoral artery (10)	6.1%	9.6%	3.5%	5.3%	−0.8%
Left profunda femoris artery (11)	5.4%	4.8%	−0.6%	5.0%	−0.4%
Left femoral artery (12)	5.5%	4.9%	−0.6%	5.1%	−0.4%
Branches of the abdominal aorta					
Inferior mesenteric artery (13)	0.9%	0.9%	0.0%	0.5%	−0.4%
Superior mesenteric artery (14)	11.0%	10.4%	−0.6%	10.9%	−0.1%
Celiac trunk (15)	15.4%	14.8%	−0.6%	15.5%	0.1%
Left renal artery (16)	9.5%	9.5%	0.0%	10.0%	0.5%
Right renal artery (17)	9.3%	9.5%	0.2%	10.0%	0.7%

LVAD, left ventricular assistance device; CO, cardiac output.

a The exact locations of the aortic branches are displayed in Figure 4E with corresponding labels in the table.

**Table 2:** Flow divisions in aorta branches with and without LVAD, considering femoral and axillary perfusion at 60% of the total flow.

Branch of the aorta^a^	Without LVAD	LVAD via femoral artery perfusion		LVAD via axillary artery perfusion	
	Flow division (%)	Flow division (%)	Variation (%)	Flow division (%)	Variation (%)
Input					
LVAD inflow (1)	0%	24.7%	-	24.7%	-
CO (2)	100%	75.3%	-	75.3%	-
Output					
Upper extremity arteries and carotid arteries					
Right subclavian artery (3)	3.0%	2.6%	−0.4%	3.7%	0.7%
Right common carotid artery (4)	7.3%	6.6%	−0.7%	7.1%	−0.2%
Left common carotid artery (5)	4.3%	4.2%	−0.1%	4.9%	0.6%
Left subclavian artery (6)	9.2%	9.0%	−0.2%	10.3%	1.1%
Lower extremity arteries					
Right internal iliac artery (7)	3.9%	3.2%	−0.7%	3.7%	−0.2%
Left internal iliac artery (8)	3.9%	3.3%	−0.6%	3.7%	−0.3%
Right profunda femoris artery (9)	5.3%	4.4%	−0.9%	4.7%	−0.6%
Right femoral artery (10)	6.1%	15.3%	9.2%	5.0%	−1.1%
Left profunda femoris artery (11)	5.4%	4.3%	−1.1%	4.7%	−0.7%
Left femoral artery (12)	5.5%	4.4%	−1.1%	4.8%	−0.7%
Branches of the abdominal aorta					
Inferior mesenteric artery (13)	0.9%	0.8%	−0.1%	0.9%	0.0%
Superior mesenteric artery (14)	11.0%	9.6%	−1.4%	10.7%	−0.3%
Celiac trunk (15)	15.4%	13.9%	−1.5%	15.5%	0.1%
Left renal artery (16)	9.5%	9.1%	−0.4%	10.2%	0.7%
Right renal artery (17)	9.3%	9.3%	0.0%	10.3%	1.0%

LVAD, left ventricular assistance devices; CO, cardiac output.

a The exact locations of the aortic branches are displayed in Figure 4E with corresponding labels in the table.

### Boundary conditions

A typical ascending aorta blood flow waveform corresponding to the normal time-average CO, which is assumed to be 5 L/min in this work, was imposed at the inlet, as shown in Figure 1A. The Windkessel models were applied to outlets to represent the impedance of the distal vascular bed to blood flow, as shown in Figure 2 and Supplementary Figure S3.

The LVAD pump function was simplified as a graft inlet with a constant flow rate. Two LVAD support configurations were modeled by introducing a graft at either the FA or the right AA. For each configuration, two constant LVAD flow rates were simulated: 2 L/min and 3 L/min. These were chosen to represent scenarios where the native cardiac output is, respectively, higher or lower than the LVAD output. A summary of the boundary conditions for the standard and LVAD-supported configurations is provided in Supplementary Table S1.

The Windkessel model parameters, calibrated for the baseline condition (without LVAD), were held constant for all simulations with LVAD support. This assumption is based on the established practice in hemodynamic simulations (15–18) and the hypothesis that the characteristics of the distal arterial system remain essentially unchanged immediately post-implantation when total perfusion is restored to a near-normal level (approximately 5 L/min) (19–21). This approach, necessitated by the lack of patient-specific postoperative data, ensures that the observed hemodynamic differences are attributable primarily to the LVAD cannulation site (FA vs. AA).

## Results

### Aortic branch perfusion estimation via CFD model

**Figure 3:**
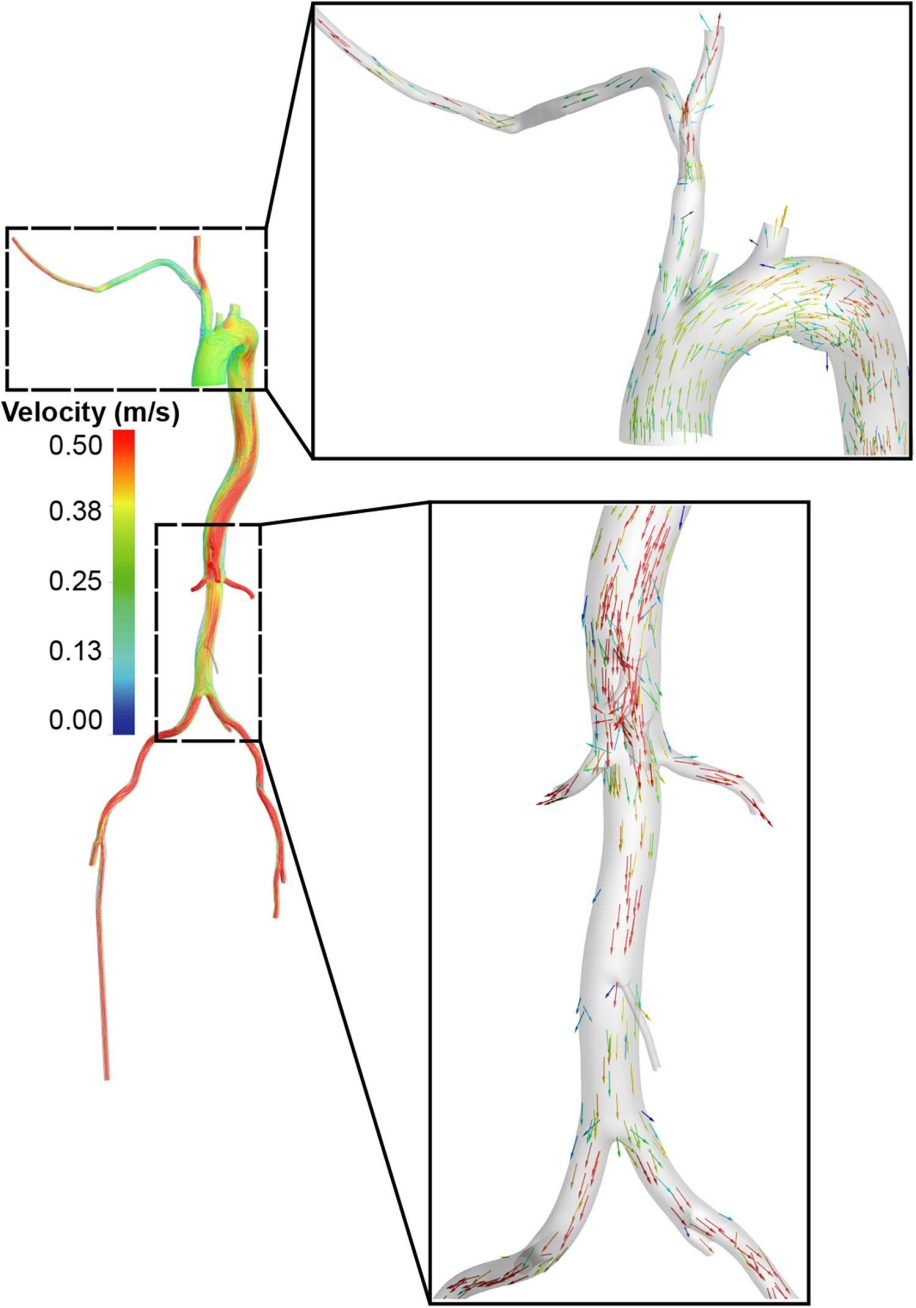
Blood flow distribution in the standard group.

**Figure 4:**
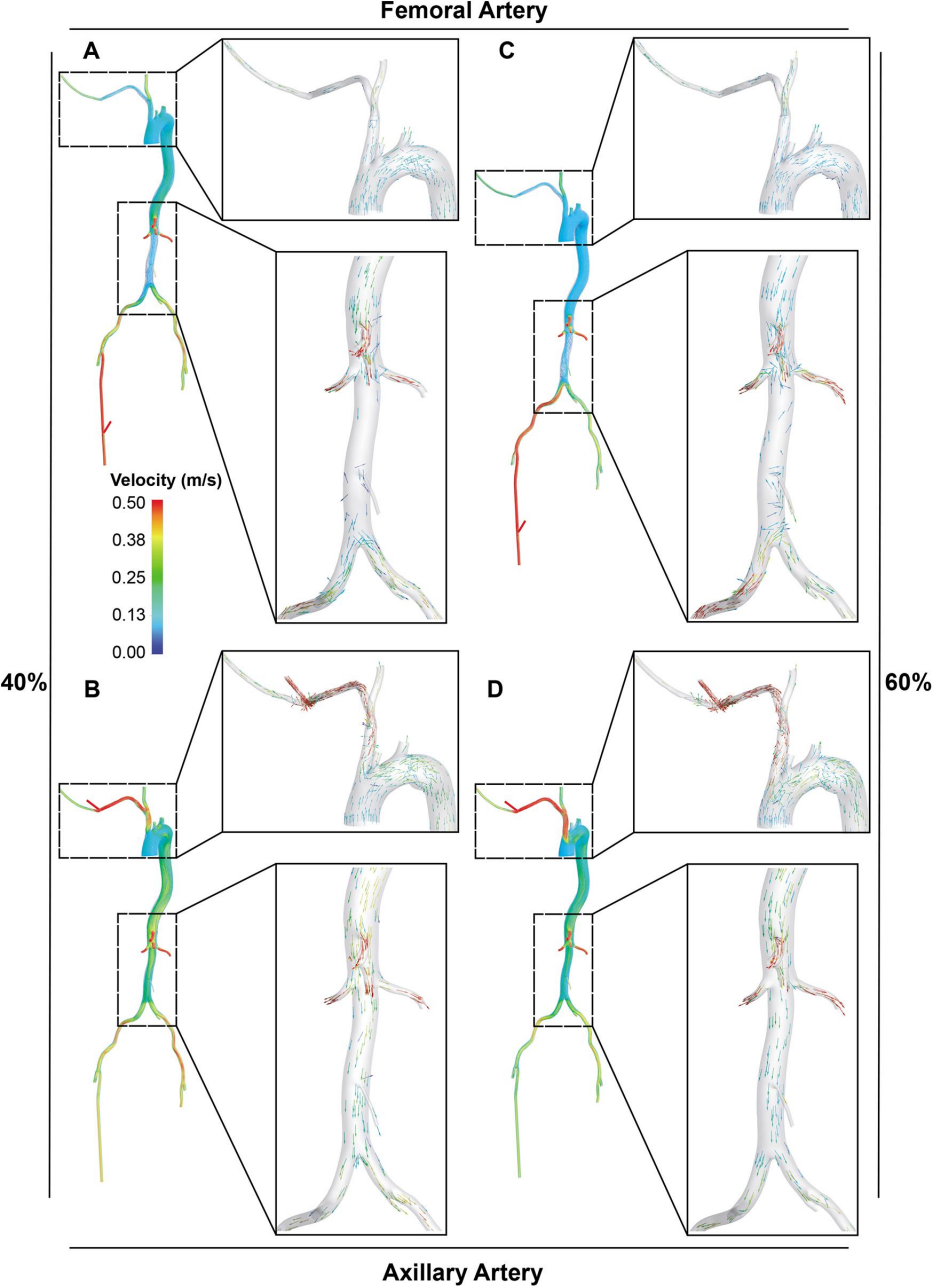
Visualization of blood flow distribution in the aorta and its main branches. A and B. With LVAD supplying 40% of total flow, the blood flow distribution in the femoral **(A)** and axillary perfusion group **(B)** is shown. C and D. With LVAD supplying 60% of total flow, blood flow distribution in the femoral **(C)** and axillary perfusion group **(D).**

The Extra-LVAD maintained a flow of 1.82-3.25 LPM for all patients, which accounted for 36%–65% of the total flow, with a CO of 5 LPM (Figure 1B). Hence, we studied the hemodynamic changes caused by the Extra-LVAD in our models under two conditions: the LVAD supplying 40% (Figure 1C) and 60% (Figure 1D) of the total flow. Cardiac ejection accounts for 75.3% of total perfusion at the end-systolic phase, with LVAD covering the remaining 24.7%. Tables 1, 2 detail the distribution of blood flow in the aortic branches, with LVAD accounting for 40% or 60% of total blood flow. Figure 2 shows the location of the main aortic branches studied by our research, including the subclavian artery, common carotid artery, internal iliac artery, profunda femoris artery, femoral artery, renal artery, inferior mesenteric artery (IMA), superior mesenteric artery (SMA), and celiac trunk. Figures 3, 4 display the flow patterns. Regardless of the amount of blood flow the LVAD provides, axillary perfusion consistently outperformed femoral perfusion in most of these arteries, except at the placement site and the inferior mesenteric artery.

For the abdominal aorta branches, axillary perfusion resulted in a significant decrease in blood supply to the IMA by 44.4% at 40% LVAD flow, but this reduction disappeared at 60% LVAD flow. At 60% LVAD flow, all arteries supplying the intestines and kidneys showed increased blood flow with axillary perfusion compared to femoral perfusion. Specifically, the IMA showed no decrease as opposed to an 11.1% reduction with femoral perfusion, the SMA decreased by 2.8% compared to a 12.7% decrease, the celiac trunk increased by 0.6% instead of decreasing by 9.7% with femoral perfusion, the left renal artery (LRA) increased by 7.4% instead of a 4.2% decrease, and the right renal artery (RRA) increased by 10.8% compared to no change. In the lower extremities, femoral perfusion also led to greater reductions in blood supply. Except for the right femoral artery as the perfusion site, at 60% LVAD flow, blood supply to all lower extremity arteries decreased by more than 15% with femoral perfusion. In comparison, they experienced less than a 13% reduction with axillary perfusion. The upper extremity and carotid artery blood flow remained stable at 40% LVAD, regardless of the perfusion approach. At 60% LVAD flow, axillary perfusion significantly increased blood flow to three of the arteries, including the right subclavian artery (23.3%), the left common carotid artery (14.0%), and the left subclavian artery (12.0%). Still, femoral perfusion decreased the blood flow in these arteries. Blood flow increased notably at the catheter insertion sites, especially the right femoral, with a 151.5% increase at 60% LVAD flow with femoral perfusion.

In terms of blood flow disorders, axillary perfusion may cause turbulent flow in the brachiocephalic trunk (Figures 4B, D), while femoral perfusion may interfere with cardiac ejection in the abdominal aorta, significantly reducing blood flow to it (Figures 4A, C).

### WSS assessment in aortic branches via CFD model

**Figure 5:**
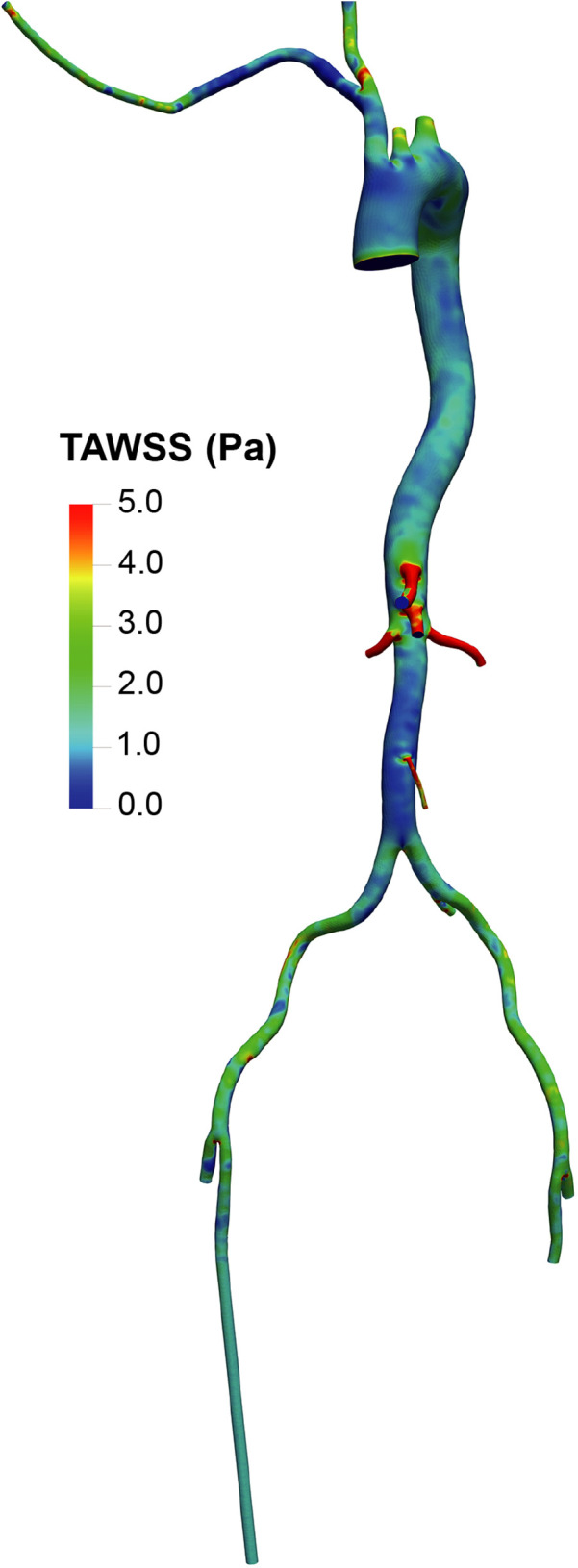
Overview of time-averaged wall shear stress (TAWSS) in the aorta and its branches in the standard group.

**Figure 6:**
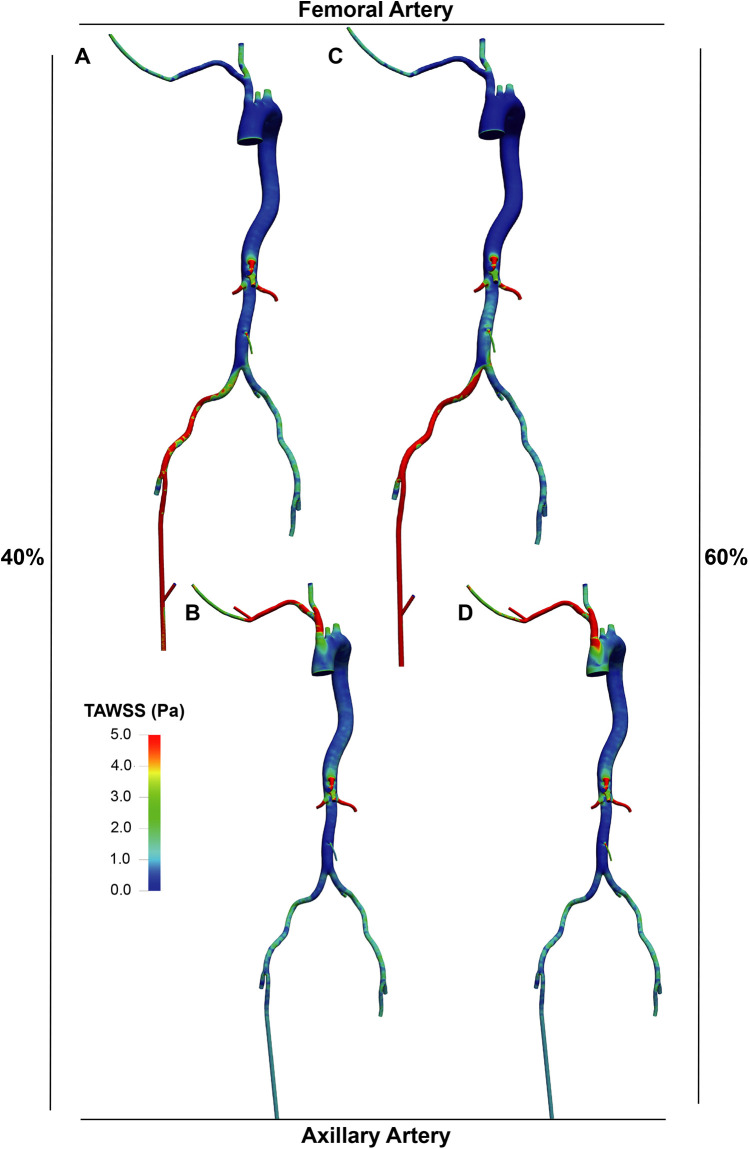
TAWSS distribution in the LVAD group. A and B. With LVAD supplying 40% of total flow, TAWSS distributions in the femoral **(A)** and axillary **(B)** perfusion groups. C and D. With LVAD supplying 60% of total flow, TAWSS distributions in the femoral **(C)** and axillary **(D)** perfusion groups.

The distribution of TAWSS (Figures 5, 6) showed values exceeding 5 Pa at all points where the perfusion graft is attached. With femoral perfusion, TAWSS in the thoracic and upper abdominal aorta above the kidneys decreased significantly to less than 1 Pa, particularly when LVAD contributed 60% of total perfusion. However, in the axillary perfusion group, the distribution of TAWSS values was more similar to that of the standard group, regardless of total perfusion.

**Figure 7:**
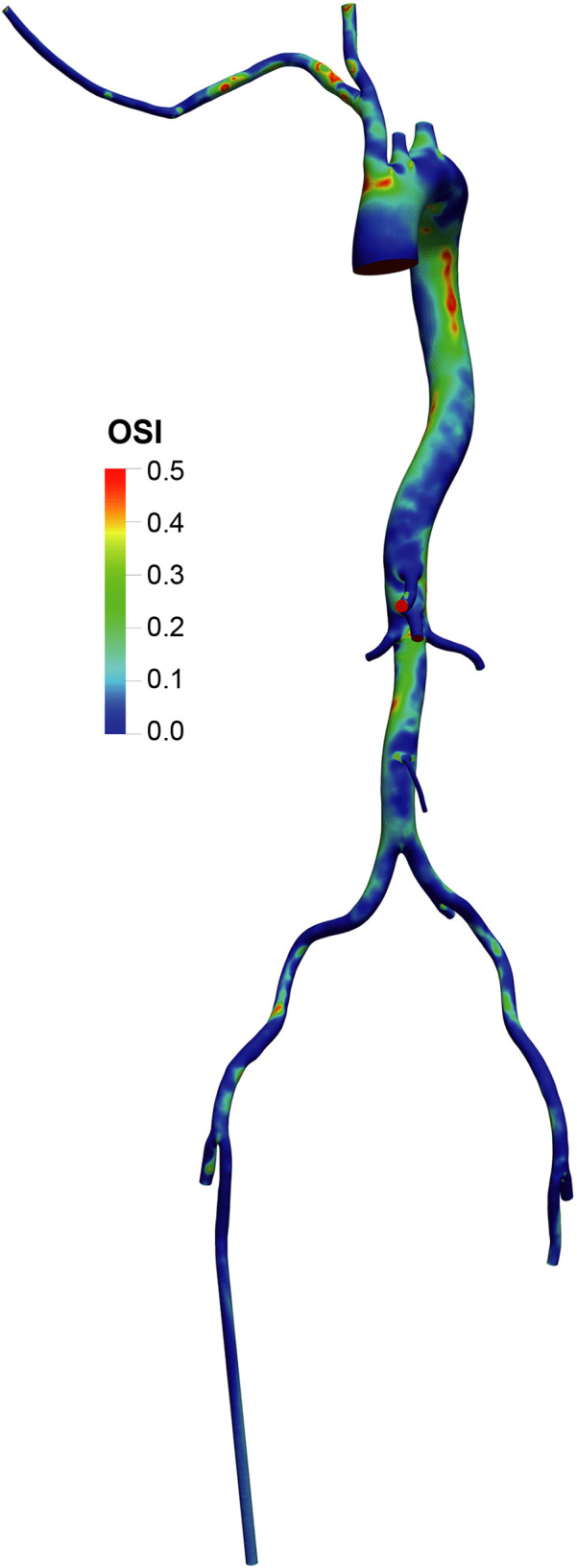
Overview of the oscillatory shear index (OSI) in the aorta and its branches in the standard group.

**Figure 8:**
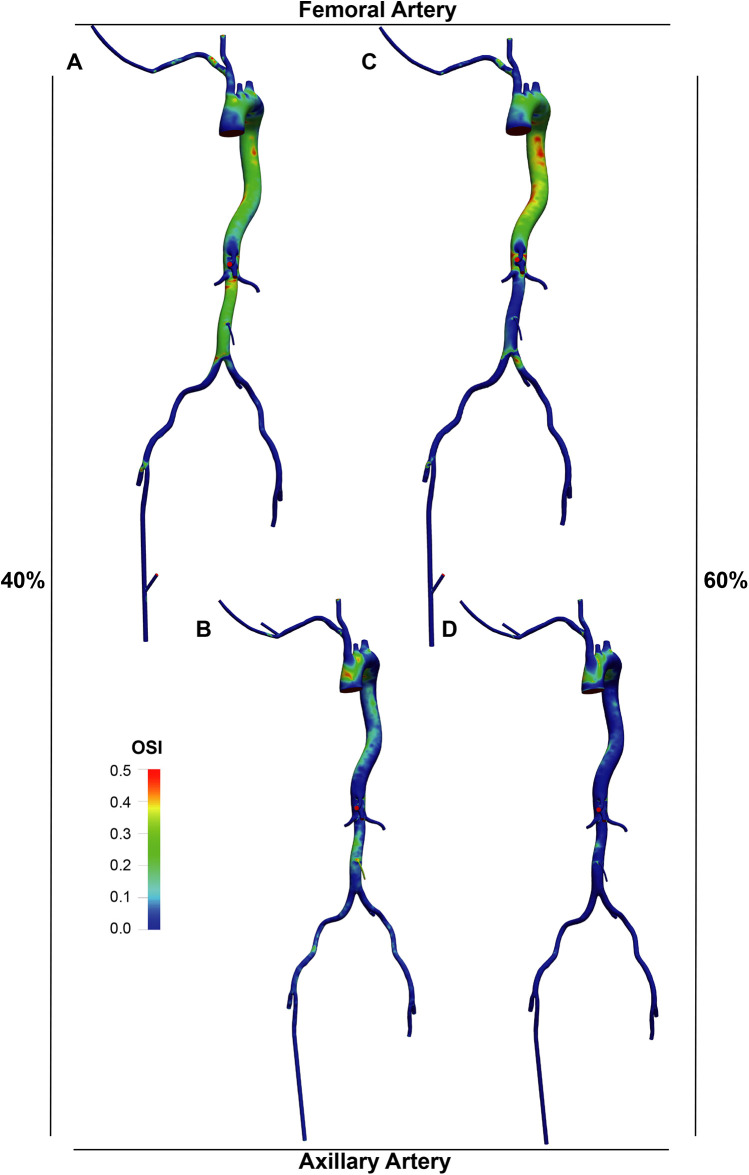
OSI distribution in the LVAD group. A and B. With LVAD supplying 40% of total flow, OSI distributions in the femoral **(A)** and axillary **(B)** perfusion groups. C and D. With LVAD supplying 60% of total flow, OSI distributions in the femoral **(C)** and axillary **(D)** perfusion groups.

The OSI distribution (Figures 7, 8) was generally lower in axillary perfusion (less than 0.2 in the most regions of the aorta) compared to femoral perfusion (more than 0.2 in the most regions), especially in the thoracic aorta and the upper abdominal aorta. This indicates less pronounced changes in WSS direction with axillary perfusion as LVAD flow increased.

## Discussion

This study has several limitations. First, the LVAD was modeled as a constant-flow inlet at 2 L/min and 3 L/min, without dynamic pump-arterial coupling. This simplification may underestimate the competitive interplay between the LVAD and the native heart, potentially leading to inaccuracies in estimated flow rates through the aortic branches. Second, as the primary objective was to compare the two perfusion sites rather than obtain precise hemodynamic values for every small branch, normalized hemodynamic waveforms were employed. Critical misrepresentation would be more likely in a pulsatile environment or with a pulsatile LVAD model, which was not the focus of this study. Furthermore, the model relies on assumptions of rigid, non-remodeling vessel walls and Newtonian blood rheology. Finally, the sample size was limited. These factors collectively differentiate our computational approach from real-world clinical studies, underscoring the need for further investigation.

From a hemodynamic perspective, we explored the pros and cons in two aspects- perfusion and WSS. Our CFD model showed that axillary perfusion resulted in less reduced perfusion in major aortic branches than femoral perfusion. This was consistent regardless of whether the LVAD provided 40% or 60% of total perfusion, except in the intubation arteries and the IMA. Perfusion to the IMA decreased significantly only at the 40% LVAD flow rate, but improved at 60% with axillary perfusion. Therefore, axillary perfusion may not critically compromise IMA perfusion, particularly given that our patients' average initial pump flow was 2.68 (±0.60) L/min (approximately 54% of a 5 L/min CO). Regarding hemodynamic complications, specifically hyperperfusion syndrome (22), our model suggests that the axillary approach may not increase its incidence. Hyperperfusion syndrome is typically associated with over a 100% increase in carotid perfusion (23, 24). The axillary approach resulted in only a 13% increase in carotid artery flow, which may be insufficient to precipitate the syndrome. Conversely, the 24% increase in flow observed in the distal right axillary artery may be clinically relevant, potentially explaining patient reports of arm swelling and numbness.

For the WSS aspect, with femoral perfusion, the TAWSS significantly decreased, with its OSI increased, indicating prevalent aortic blood flow disturbances. However, in the axillary perfusion group, because it provides perfusion in the same direction as the heart, the OSI was generally lower, except at the ascending aorta, and the reduction in TAWSS was less marked. High WSS is usually beneficial, whereas low WSS might be associated with high OSI due to flow reversal (8, 25) and could lead to conditions such as atherosclerosis (8, 26) and aortic or arterial aneurysms (27, 28), possibly due to drastic fluctuations. Based on a CDF model established by our patient's CTA, the axillary approach may offer improved hemodynamics compared to the conventional femoral approach.

## Conclusions

We developed a CFD model using patient CTA data to analyze key fluid parameters. Our findings suggest that, within the constraints of a model assuming rigid vessel walls and Newtonian blood flow, the axillary approach may improve overall circulation. It is associated with a more favorable hemodynamic profile, including enhanced perfusion to abdominal aortic branches and more stable flow patterns, compared to the traditional femoral approach. The jugular-axillary approach thus emerges as a promising configuration for future percutaneous extra-LVAD procedures. Further validation through real-world clinical studies is warranted.

## Data Availability

The original contributions presented in the study are included in the article/Supplementary Material, further inquiries can be directed to the corresponding author/s.

## Supplementary material

Supplementary Figure S1 Detailed mesh with boundary layers for the baseline model (without LVAD).

Supplementary Figure S2 Grid (Mesh) independence in the standard group (without LVAD). A. The graph (A) plots the computed TAWSS against the total number of grid elements for three systematically refined meshes. TAWSS values are presented for two different surface domains: full wall (B) and clip wall (C). B and C. The orange-shaded regions on the 3D aorta schematics (insets) illustrate the respective surface domains from which the TAWSS values were extracted: Full wall (B), the entire luminal surface of the aortic model, including all major branches. Clip Wall (C), a truncated aortic wall, created by clipping the inlet, outlets, and branch vessels, retaining only the primary aortic trunk to minimize the influence of local flow effects at the boundaries.

Supplementary Figure S3 The schematic of inlet/outlet boundary conditions and LVAD graft locations.

## References

[1] Moonsamy P Axtell AL Ibrahim NE Funamoto M Tolis G Lewis GD Survival after heart transplantation in patients bridged with mechanical circulatory support. J Am Coll Cardiol. (2020) 75:2892–905. 10.1016/j.jacc.2020.04.037 32527398

[2] Trivedi JR Cheng A Singh R Williams ML Slaughter MS. Survival on the heart transplant waiting list: impact of continuous flow left ventricular assist device as bridge to transplant. Ann Thorac Surg. (2014) 98:830–4. 10.1016/j.athoracsur.2014.05.019 25087934

[3] Rihal CS Naidu SS Givertz MM Szeto WY Burke JA Kapur NK 2015 SCAI/ACC/HFSA/STS clinical expert consensus statement on the use of percutaneous mechanical circulatory support devices in cardiovascular care: endorsed by the American Heart Assocation, the Cardiological Society of India, and Sociedad Latino Americana de Cardiologia Intervencion; Affirmation of Value by the Canadian Association of Interventional Cardiology-Association Canadienne de Cardiologie d'intervention. J Am Coll Cardiol. (2015) 65:e7–e26. 10.1016/j.jacc.2015.03.036 25861963

[4] Li P Zhang X Chen S Hsu P Wu T Qian S Case report: successful percutaneous extracorporeal magnetic levitation ventricular assist device support in a patient with left heart failure due to dilated cardiomyopathy. Front Cardiovasc Med. (2023) 10:1093794. 10.3389/fcvm.2023.1093794 36742072 PMC9892048

[5] Lewin D Nersesian G Lanmüller P Schoenrath F Falk V Potapov EV Complications related to the access site after transaxillary implantation of a microaxial left ventricular assist device. J Heart Lung Transplant. (2023) 42:679–87. 10.1016/j.healun.2022.12.018 36653272

[6] El-Sayed Ahmed MM Aftab M Singh SK Mallidi HR Frazier OH. Left ventricular assist device outflow graft: alternative sites. Ann Cardiothorac Surg. (2014) 3:541–5. 10.3978/j.issn.2225-319X.2014.09.03 25452918 PMC4229468

[7] Schmack L Ali-Hasan-Al-Saegh S Weymann A Pizanis N Akhyari P Zubarevich A Inflammatory and hemolytic responses of microaxial flow pump temporary ventricular assist devices via axillary access in cardiogenic shock. Medicina (Kaunas). (2024) 60(12):1960. 10.3390/medicina60121960 39768841 PMC11677742

[8] Peiffer V Sherwin SJ Weinberg PD. Does low and oscillatory wall shear stress correlate spatially with early atherosclerosis? A systematic review. Cardiovasc Res. (2013) 99:242–50. 10.1093/cvr/cvt044 23459102 PMC3695746

[9] Shaaban AM Duerinckx AJ. Wall shear stress and early atherosclerosis: a review. AJR Am J Roentgenol. (2000) 174:1657–65. 10.2214/ajr.174.6.1741657 10845502

[10] Chatzizisis YS Coskun AU Jonas M Edelman ER Feldman CL Stone PH. Role of endothelial shear stress in the natural history of coronary atherosclerosis and vascular remodeling: molecular, cellular, and vascular behavior. J Am Coll Cardiol. (2007) 49:2379–93. 10.1016/j.jacc.2007.02.059 17599600

[11] Menter FR Kuntz M Langtry R. Ten years of industrial experience with the SST turbulence model. Turbul Heat Mass Transfer. (2003) 4:625–32.

[12] Fraser KH Taskin ME Griffith BP Wu ZJ. The use of computational fluid dynamics in the development of ventricular assist devices. Med Eng Phys. (2011) 33:263–80. 10.1016/j.medengphy.2010.10.014 21075669 PMC3053072

[13] Alastruey J Xiao N Fok H Schaeffter T Figueroa CA. On the impact of modelling assumptions in multi-scale, subject-specific models of aortic haemodynamics. J R Soc Interface. (2016) 13(119):20160073. 10.1098/rsif.2016.0073 27307511 PMC4938079

[14] Westerhof N Bosman F De Vries CJ Noordergraaf A. Analog studies of the human systemic arterial tree. J Biomech. (1969) 2(2):121–43. 10.1016/0021-9290(69)90024-4 16335097

[15] Karmonik C Partovi S Loebe M Schmack B Weymann A Lumsden AB Computational fluid dynamics in patients with continuous-flow left ventricular assist device support show hemodynamic alterations in the ascending aorta. J Thorac Cardiovasc Surg. (2014) 147:1326–33.e1321. 10.1016/j.jtcvs.2013.09.069 24345553

[16] Li L Shi L Tan X Zhao Y. Influence of LVAD cannula outflow graft flow rate and location on fluid-particle interactions and thrombi distribution: a primary numerical study. J Cardiovasc Transl Res. (2024) 17:1316–27. 10.1007/s12265-024-10547-1 39039390 PMC11634971

[17] Black SM. Patient-specific multi-dimensional CFD simulations based on 4D Flow-MRI for the haemodynamic assessment of aortic dissections and perfusion optimisation of vascular grafts. (2024).

[18] Black MS Maclean C Barrientos PH Ritos K McQueen A Kazakidi A. Calibration of patient-specific boundary conditions for coupled CFD models of the aorta derived from 4D flow-MRI. Front Bioeng Biotechnol. (2023) 11:1178483. 10.3389/fbioe.2023.1178483 37251565 PMC10210162

[19] Salvi L Alfonsi J Grillo A Pini A Soranna D Zambon A Postoperative and mid-term hemodynamic changes after replacement of the ascending aorta. J Thorac Cardiovasc Surg. (2022) 163:1283–92. 10.1016/j.jtcvs.2020.05.031 32624310

[20] Imamura T Jeevanandam V Kim G Raikhelkar J Sarswat N Kalantari S Optimal hemodynamics during left ventricular assist device support are associated with reduced readmission rates. Circ Heart Fail. (2019) 12:e005094. 10.1161/circheartfailure.118.005094 30704291 PMC7039343

[21] Symons JD Deeter L Deeter N Bonn T Cho JM Ferrin P Effect of continuous-flow left ventricular assist device support on coronary artery endothelial function in ischemic and nonischemic cardiomyopathy. Circ Heart Fail. (2019) 12:e006085. 10.1161/circheartfailure.119.006085 31422672

[22] Lietz K Brown K Ali SS Colvin-Adams M Boyle AJ. Anderson D The role of cerebral hyperperfusion in postoperative neurologic dysfunction after left ventricular assist device implantation for end-stage heart failure. J Thorac Cardiovasc Surg. (2009) 137:1012–9. 10.1016/j.jtcvs.2008.11.034 19327532

[23] van Mook WNKA Rennenberg RJMW Schurink GW van Oostenbrugge RJ Mess WH Hofman PAM Cerebral hyperperfusion syndrome. Lancet Neurol. (2005) 4:877–88. 10.1016/s1474-4422(05)70251-9 16297845

[24] Ascher E Markevich N Schutzer RW Kallakuri S Jacob T Hingorani AP. Cerebral hyperperfusion syndrome after carotid endarterectomy: predictive factors and hemodynamic changes. J Vasc Surg. (2003) 37:769–77. 10.1067/mva.2003.231 12663976

[25] Ku DN Giddens DP Zarins CK Glagov S. Pulsatile flow and atherosclerosis in the human carotid bifurcation. Positive correlation between plaque location and low oscillating shear stress. Arteriosclerosis. (1985) 5:293–302. 10.1161/01.atv.5.3.293 3994585

[26] Stone PH Coskun AU Kinlay S Popma JJ Sonka M Wahle A Regions of low endothelial shear stress are the sites where coronary plaque progresses and vascular remodelling occurs in humans: an *in vivo* serial study. Eur Heart J. (2007) 28:705–10. 10.1093/eurheartj/ehl575 17347172

[27] Trenti C Ziegler M BjarnegÃ N Ebbers T Lindenberger M Dyverfeldt P Wall shear stress and relative residence time as potential risk factors for abdominal aortic aneurysms in males: a 4D flow cardiovascular magnetic resonance case-control study. J Cardiovasc Magn Reson. (2022) 24:18. 10.1186/s12968-022-00848-2 35303893 PMC8932193

[28] Xiang J Siddiqui AH Meng H. The effect of inlet waveforms on computational hemodynamics of patient-specific intracranial aneurysms. J Biomech. (2014) 47:3882–90. 10.1016/j.jbiomech.2014.09.034 25446264 PMC4261154

