# Supplementary material for: Computational hemodynamic assessment of axillary and femoral artery perfusion for extracorporeal left ventricular assist device
Source: Front Cardiovasc Med. 2025 Dec 2;12:1631144. doi: 10.3389/fcvm.2025.1631144 (PMC12706660; doi:10.3389/fcvm.2025.1631144)
Supplement: Supplementary file 1 [file Table1.docx]

**Table S1. The parameters of the inlets and outlets at each branch of the standard and two placements of LVAD in different physiological drive.**

| **Type** | | | **Inlet** | **Outlet** |
| --- | --- | --- | --- | --- |
| Standard | Normal | AAO | CO_average_ =5 L/min | Windkessel |
| Axillary artery perfusion | Low perfusion | AAO | CO_average_ = 3 L/min | Windkessel |
|  |  | LVAD | LVAD=2 L/min |  |
|  | High perfusion | AAO | CO_average_ =2 L/min |  |
|  |  | LVAD | LVAD=3 L/min |  |
| Femoral artery perfusion | Low perfusion | AAO | CO_average_= 3 L/min | Windkessel |
|  |  | LVAD | LVAD=2 L/min |  |
|  | High perfusion | AAO | CO_average_=2 L/min |  |
|  |  | LVAD | LVAD=3 L/min |  |
