# Supplementary figures and images for: Computational hemodynamic assessment of axillary and femoral artery perfusion for extracorporeal left ventricular assist device
Source: Front Cardiovasc Med. 2025 Dec 2;12:1631144. doi: 10.3389/fcvm.2025.1631144 (PMC12706660; doi:10.3389/fcvm.2025.1631144)

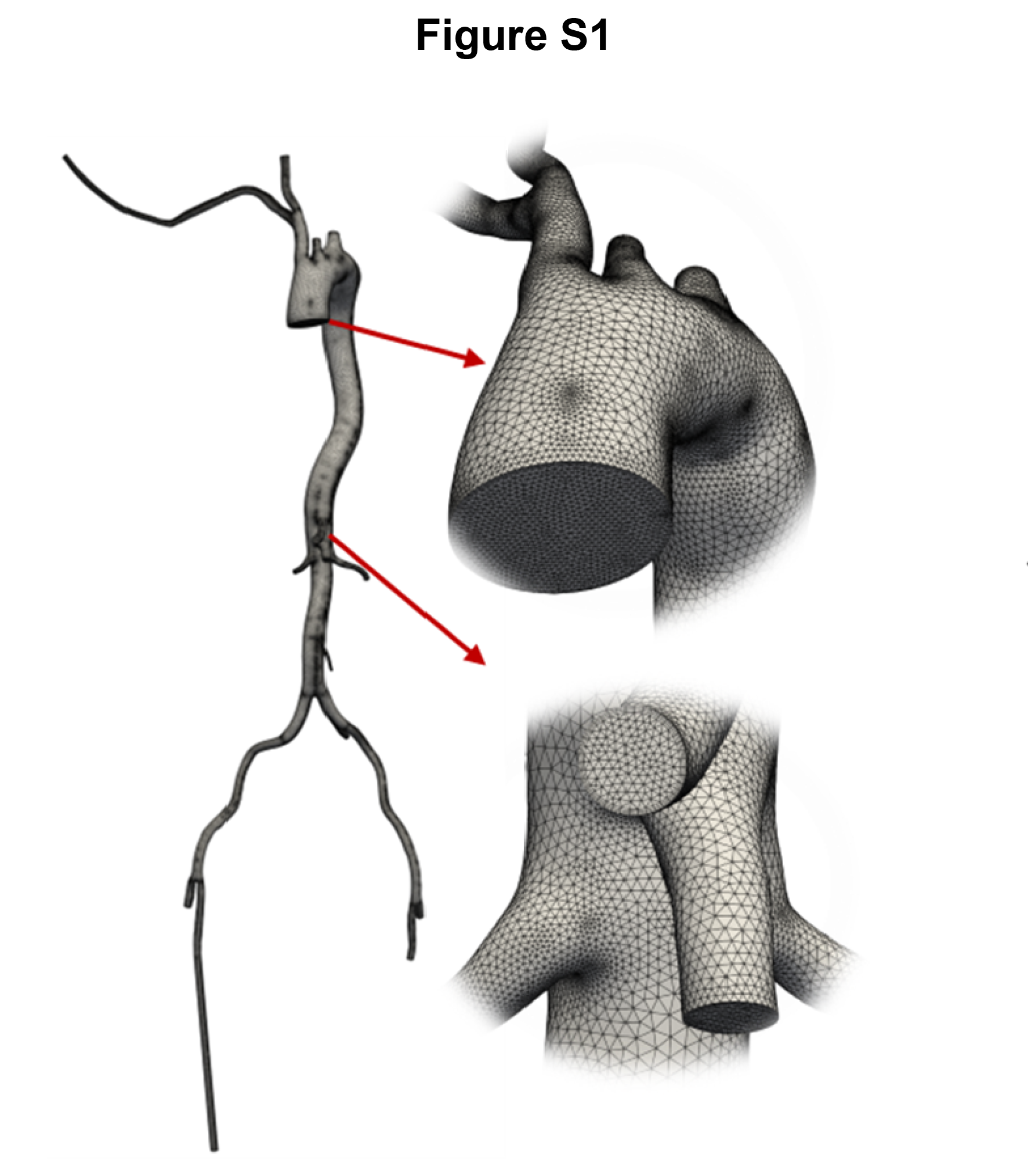

Supplement: Supplementary Figure S1 — Detailed mesh with boundary layers for the baseline model (without LVAD). [file Image1.tif]

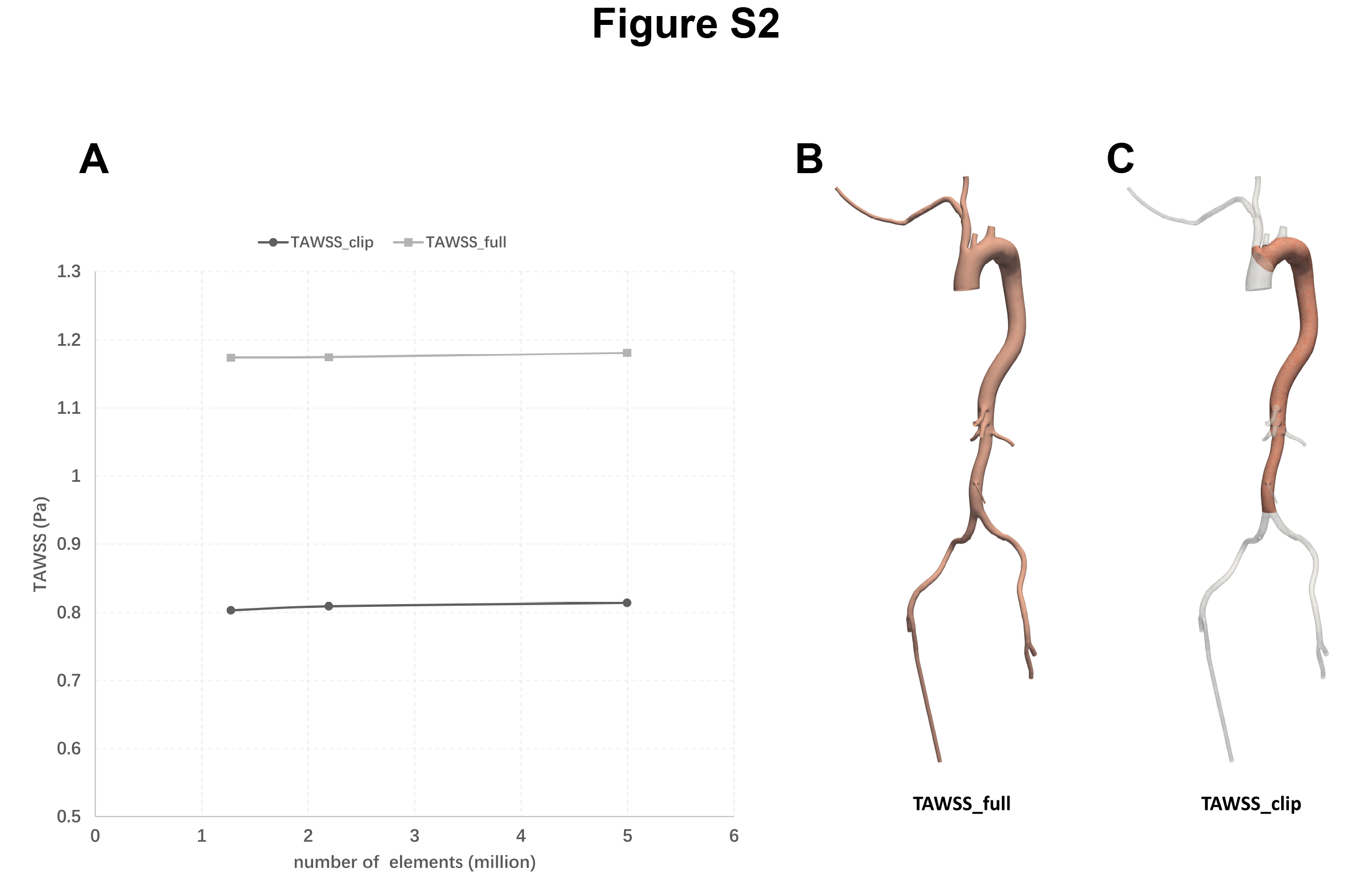

Supplement: Supplementary Figure S2 — Grid (Mesh) independence in the standard group (without LVAD). A. The graph (A) plots the computed TAWSS against the total number of grid elements for three systematically refined meshes. TAWSS values are presented for two different surface domains: full wall (B) and clip wall (C). B and C. The orange-shaded regions on the 3D aorta schematics (insets) illustrate the respective surface domains from which the TAWSS values were extracted: Full wall (B), the entire luminal surface of the aortic model, including all major branches. Clip Wall (C), a truncated aortic wall, created by clipping the inlet, outlets, and branch vessels, retaining only the primary aortic trunk to minimize the influence of local flow effects at the boundaries. [file Image2.tif]

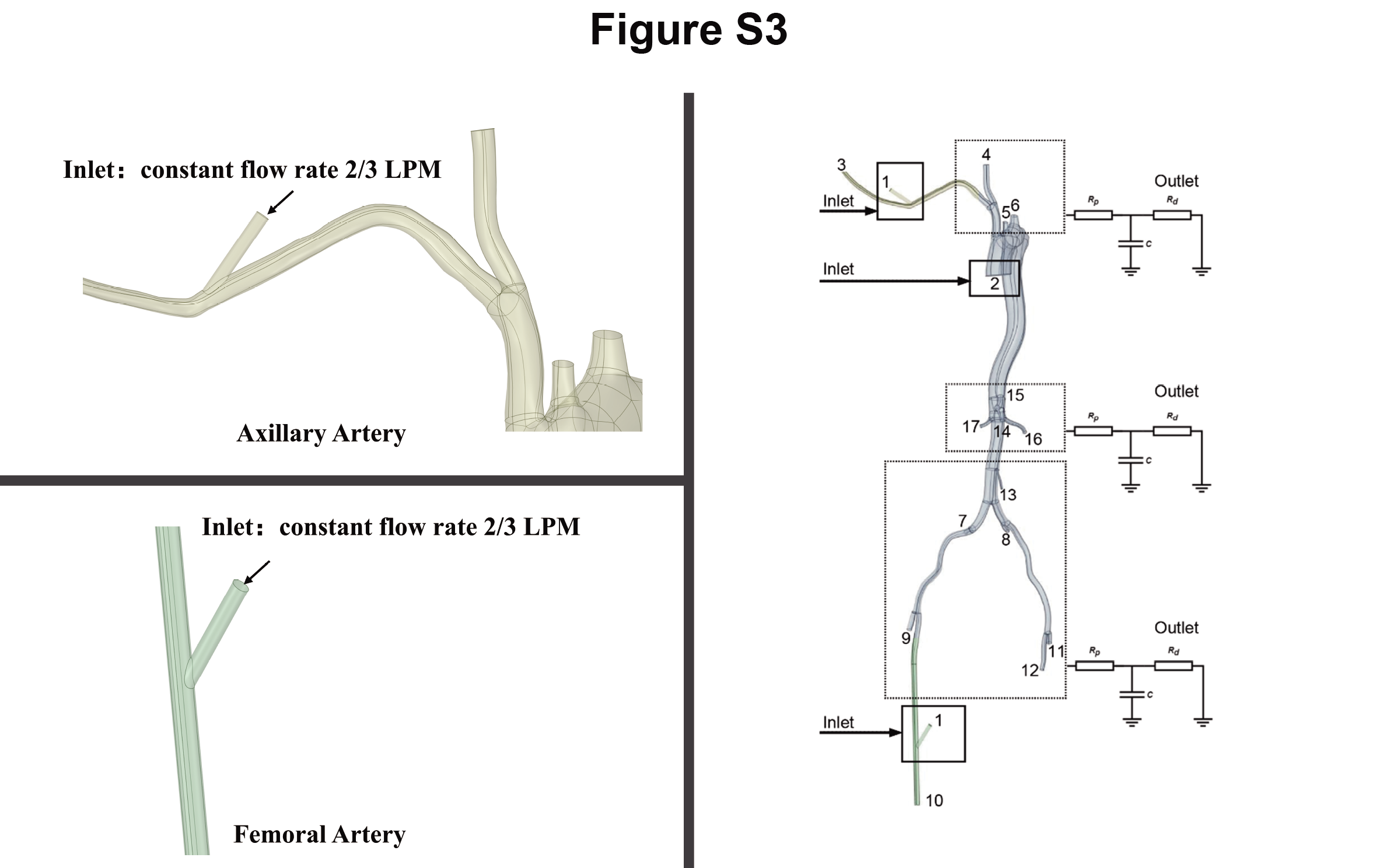

Supplement: Supplementary Figure S3 — The schematic of inlet/outlet boundary conditions and LVAD graft locations. [file Image3.tif]
